# Ca^2+^ Regulates ERp57-Calnexin Complex Formation

**DOI:** 10.3390/molecules26102853

**Published:** 2021-05-11

**Authors:** Yuya Tanikawa, Shingo Kanemura, Dai Ito, Yuxi Lin, Motonori Matsusaki, Kimiko Kuroki, Hiroshi Yamaguchi, Katsumi Maenaka, Young-Ho Lee, Kenji Inaba, Masaki Okumura

**Affiliations:** 1 School of Science and Technology, Kwansei Gakuin University, 2-1 Gakuen, Sanda 669-1337, Japan; fnc11916@kwansei.ac.jp (Y.T.); shingo.kanemura@kwansei.ac.jp (S.K.); hiroshi@kwansei.ac.jp (H.Y.); 2 Frontier Research Institute for Interdisciplinary Sciences, Tohoku University, 6-3 Aramakiaza Aoba, Aoba-ku, Sendai 980-8578, Japan; matsusaki@tokushima-u.ac.jp; 3 Department of Brain and Cognitive Science, Daegu Gyeongbuk Institute of Science and Technology, 333 Techno Jungang Daero, Daegu 42988, Korea; dai.ito.osaka@gmail.com; 4 Research Center for Bioconvergence Analysis, Korea Basic Science Institute, 162 Yeongudanji-ro, Ochang, Cheongju 28119, Korea; linyuxi@kbsi.re.kr (Y.L.); mr0505@kbsi.re.kr (Y.-H.L.); 5 Institute of Advanced Medical Sciences, Tokushima University, 3-18-15 Kuramoto-cho, Tokushima 770-8503, Japan; 6 Laboratory of Biomolecular Science, Faculty of Pharmaceutical Sciences, Hokkaido University, Nishi 6, Kita 12, Kita-ku, Sapporo 060-0812, Japan; k-kimiko@pharm.hokudai.ac.jp (K.K.); maenaka@pharm.hokudai.ac.jp (K.M.); 7 Center for Research and Education on Drug Discovery, Faculty of Pharmaceutical Sciences and Global Station for Biosurfaces and Drug Discovery, Hokkaido University, Nishi 6, Kita 12, Kita-ku, Sapporo 060-0812, Japan; 8 Research Headquarters, Korea Brain Research Institute, 61 Cheomdan-ro, Dong-gu, Daegu 41068, Korea; 9 Bio-Analytical Science, University of Science and Technology, 217 Gajeong-ro, Yuseong-gu, Daejeon 34113, Korea; 10 Graduate School of Analytical Science and Technology, Chungnam National University, 99 Daehak-ro, Yuseong-gu, Daejeon 34134, Korea; 11 Institute of Multidisciplinary Research for Advanced Materials, Tohoku University, 2-1-1 Katahira, Aoba-ku, Sendai 980-8577, Japan; kenji.inaba.a1@tohoku.ac.jp; 12 Fusion Oriented Research for Disruptive Science and Technology, Japan Science Technology Agency, Chiyoda-ku, Tokyo 102-0075, Japan

**Keywords:** endoplasmic reticulum, oxidative folding, chaperone, calnexin, ERp57, human leukocyte antigen, Ca^2+^

## Abstract

ERp57, a member of the protein disulfide isomerase family, is a ubiquitous disulfide catalyst that functions in the oxidative folding of various clients in the mammalian endoplasmic reticulum (ER). In concert with ER lectin-like chaperones calnexin and calreticulin (CNX/CRT), ERp57 functions in virtually all folding stages from co-translation to post-translation, and thus plays a critical role in maintaining protein homeostasis, with direct implication for pathology. Here, we present mechanisms by which Ca^2+^ regulates the formation of the ERp57-calnexin complex. Biochemical and isothermal titration calorimetry analyses revealed that ERp57 strongly interacts with CNX via a non-covalent bond in the absence of Ca^2+^. The ERp57-CNX complex not only promoted the oxidative folding of human leukocyte antigen heavy chains, but also inhibited client aggregation. These results suggest that this complex performs both enzymatic and chaperoning functions under abnormal physiological conditions, such as Ca^2+^ depletion, to effectively guide proper oxidative protein folding. The findings shed light on the molecular mechanisms underpinning crosstalk between the chaperone network and Ca^2+^.

## 1. Introduction

The endoplasmic reticulum (ER) harbors specific enzymes and associated factors that both assist productive protein folding and eliminate the risk of protein aggregation to ensure protein homeostasis in the ER [1,2,3,4]. More than 20 protein disulfide isomerase family members (PDIs) and ER-resident oxidoreductases are believed to catalyze the oxidative folding of a wide variety of secretory and membrane proteins including insulin, immunoglobulins, and human leukocyte antigen (HLA). Most PDIs contain the Cys-X-X-Cys motif within the redox active site of their thioredoxin (Trx)-like domain(s) that catalyze disulfide introduction, isomerization, and reduction in substrates [5]. Canonical PDI consists of four Trx-like domains, the first and last of which contain the Cys-X-X-Cys motifs, forming an overall U-shaped structure [6]. Regarding the client recruiting mechanism, cutting-edge technology in the form of time-resolved single-molecule observations by high-speed atomic force microscopy demonstrated that PDI displays striking redox-dependent conformational dynamics, and consequently assembles to form a face-to-face dimer with a central hydrophobic cavity during the early folding pathway of clients, resulting in an efficient protein folding apparatus with an increased catalytic rate [7]. Unlike the substrate recognition mechanism of PDI, several solvent-exposed redox active sites of ERp46 and P5 are dedicated to rapid but promiscuous disulfide introduction [8,9,10]. Clients with multiple disulfide bonds are likely to have complicated oxidative folding pathways, hence differences in client recognition mechanisms by PDIs might be indispensable for dealing with various conformers and folding states [11].

Like PDI, ERp57 adopts an overall U-shaped Trx-like domain arrangement [12]. However, ERp57 is much less capable of introducing native disulfide bonds into reduced and denatured clients [8]. It is well known that ERp57 acts on unfolded glyco-proteins in concert with ER lectin chaperones calnexin (CNX) and calreticulin (CRT) via interaction with the ERp57 **b’** domain [13,14,15,16,17]. Nuclear magnetic resonance and isothermal titration calorimetry (ITC) studies showed that the **b’** domain is responsible for the interaction with the arm-like P-domain of CNX/CRT, in which positively charged Lys214, Lys274, and Arg282 engage in electrostatic interactions with a negatively charged tip of the arm-like P-domain in CNX/CRT [18,19]. In line with this, the ERp57-CNX/CRT complex is intimately involved in the folding and assembly of the glycosylated HLA, which is composed of heavy and light chains (β_2_-microglobulin; β2m) [20,21], and peptide loading complex (PLC) [22]. The newly synthesized HLA heavy chain (HC) interacts with the CNX-ERp57 complex during the early folding step, and natively folded HLA-HC interacts with the CRT-ERp57 complex for the assembly of HLA heterodimer with β2m and PLC [21]. Thus, several lines of evidence indicate that complex formation between ERp57 and CNX/CRT plays essential roles in oxidative folding of glycoproteins, thereby ensuring the formation of correctly-folded HLA heterodimer.

In addition to enzymatic functions, several ER-resident proteins including PDI [23], P5 [10], and CNX bind to Ca^2+^ [24], which is stored at high concentration (~1 mM) in the ER [25]. The chaperoning functions of PDI and P5 are negatively regulated by Ca^2+^, although the impact of Ca^2+^ on ERp57 is still unclear. Interestingly, CNX, a Ca^2+^-binding chaperone, has lower chaperone activity toward glycosylated and non-glycosylated HLA-HC in the presence of Ca^2+^ due to Ca^2+^-dependent conformational changes of CNX, suggesting that Ca^2+^ mediates chaperone functions in the ER [26]. Even though interactions between PDI/ERp57 and CRT are modulated by Ca^2+^ [27], the functional relationship between ER-resident chaperone networks and Ca^2+^ remains poorly understood.

Here, we performed extensive biochemical analyses of complex formation between ERp57 and the ER-luminal domain of CNX, and revealed that ERp57 strongly interacts with CNX via a non-covalent bond in the absence of Ca^2+^. The ERp57-CNX complex not only promotes the oxidative folding of HLA-HC, but also inhibits client aggregation. These findings shed light on the molecular mechanism underpinning crosstalk in chaperone-regulated protein homeostasis and Ca^2+^.

## 2. Results

### 2.1. Complex Formation between ERp57 and CNX Is Modulated by Ca^2+^

**Figure 1:**
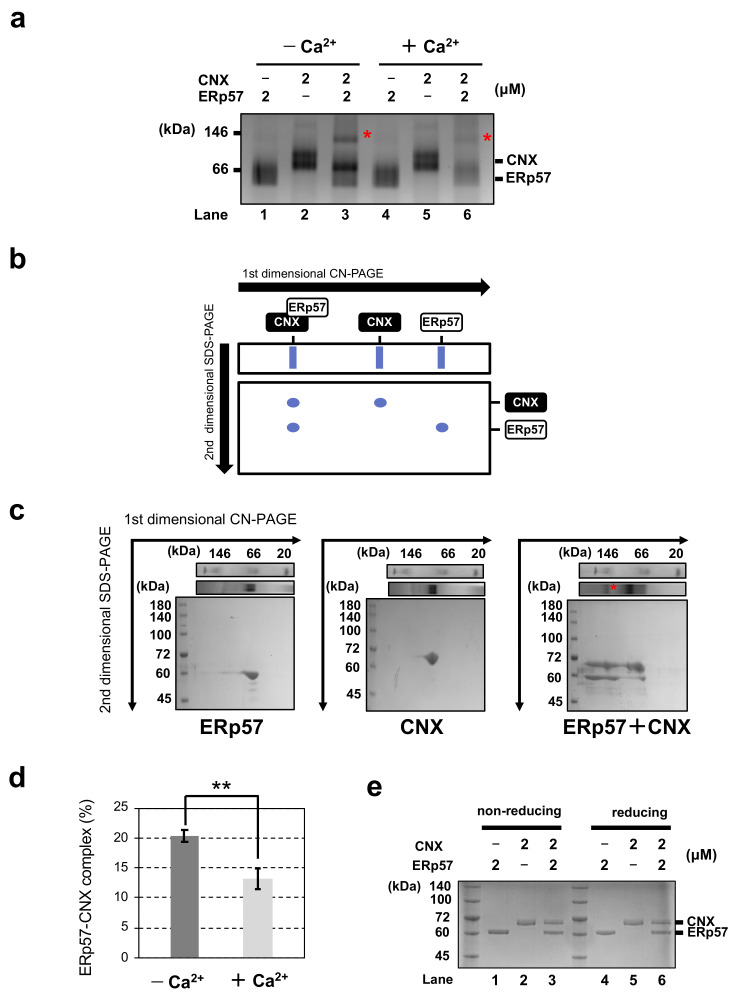
Complex formation between ERp57 and CNX is impaired by Ca^2+^. (**a**) Clear-native PAGE of ERp57 and CNX in the presence or absence of Ca^2+^. (**b**) Schematic representation of two-dimensional PAGE. (**c**) Gel images of two-dimensional PAGE in Figure 1a, lanes 1, 2, and 3. (**d**) Quantification and statistical analysis of the fraction of the ERp57-CNX complex shown in Figure 1a, lanes 3 and 6 (*n* = 3, mean ± SD). ** *p* < 0.01. (**e**) Non-reducing and reducing SDS-PAGE of ERp57 and CNX without Ca^2+^. Experiments were independently repeated three times with reproducible results.

To probe complex formation between ERp57 and CNX, we mixed 2 μM ERp57 with 2 μM of the luminal domain of CNX and analyzed their oligomeric states using clear-native polyacrylamide gel electrophoresis (CN-PAGE; Figure 1a). CN-PAGE can detect non-covalent interactions between complexes under native (non-denatured) conditions [28]. Although the two bands around 66 kDa and 57 kDa are consistent with monomeric CNX and ERp57, respectively, the upper band around 146 kDa (marked with an asterisk) was observed in the presence of both proteins (Figure 1a, lanes 3 and 6). To further characterize the upper band, two-dimensional PAGE (2D-PAGE) was employed to separate proteins with different molecular weights (Figure 1b). The first (one-dimensional PAGE; 1D-PAGE) separation step was performed using the same conditions as in Figure 1a, and gel strips were then separated in the presence of sodium dodecyl sulfate (SDS) and 2-mercaptoethanol in the second (2D-PAGE) step (Figure 1c). Bands corresponding to ERp57 (Figure 1c, left) and CNX (Figure 1c, middle) in 1D-PAGE were clearly detected as single spots in 2D-PAGE, while the band marked by an asterisk was divided into two spots corresponding to CNX and ERp57 (Figure 1c, right), indicating complex formation between ERp57 and CNX.

To investigate the impact of Ca^2+^ on the formation of the ERp57-CNX complex, we quantitatively analyzed the amount of complex using CN-PAGE with or without Ca^2+^. The amount of ERp57-CNX complex significantly increased in the absence of Ca^2+^ (Figure 1a, lanes 3 and 6, Figure 1d). This is presumably because electrostatic interactions between ERp57 and CNX are impaired by Ca^2+^. To further examine whether the interaction between ERp57 and CNX is covalent or non-covalent, non-reducing and reducing sodium dodecyl sulfate-polyacrylamide gel electrophoresis (SDS-PAGE) experiments were performed with/without 5% (*v*/*v*) 2-mercaptoethanol. Unlike CN-PAGE, the band corresponding to the ERp57-CNX complex was not detected in non-reducing SDS-PAGE (Figure 1e, lanes 3 and 6). Consistent with previous studies, this result indicates that ERp57 interacts with CNX non-covalently [18,19]. These results suggest that Ca^2+^ is a modulator of ERp57-CNX complex formation in the ER.

### 2.2. Ca^2+^ Hampers the Interaction between ERp57 and CNX

**Figure 2:**
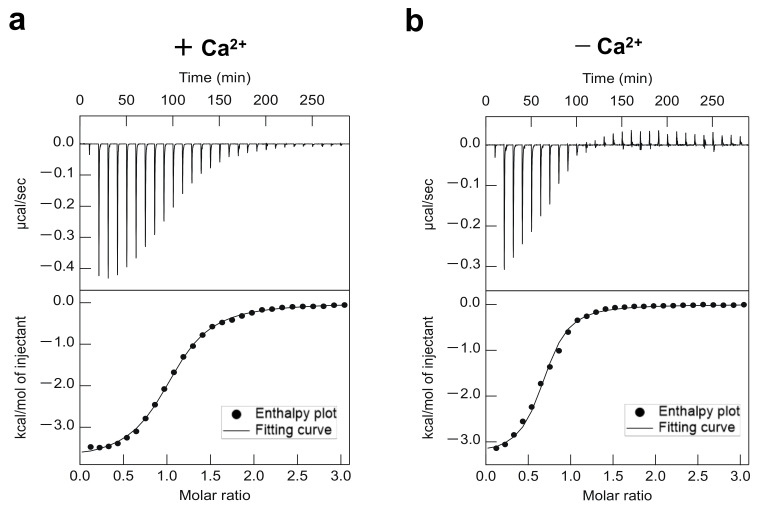
Thermodynamic characterization of effects of Ca^2+^ on the interaction between ERp57 and CNX. The ITC data show the titration of ERp57 against CNX in the presence (**a**) and absence (**b**) of Ca^2+^. Thermodynamic parameters for ERp57 binding to CNX are compiled in Table 1. The error values obtained from the model fitting were shown in Table 1.

To further explore the binding affinity between ERp57 and CNX, a set of isothermal titration calorimetry (ITC) measurements were performed with or without Ca^2+^. ERp57 in the syringe was titrated into a solution containing CNX in the cell. The ITC thermograms of the interaction between ERp57 and CNX in the presence or absence of Ca^2+^ revealed an exothermic reaction, which reflects favorable van der Waals, hydrogen bonding, and electrostatic interactions (Figure 2). Thermodynamic analyses by ITC provided quantitative information on the interaction between ERp57 and CNX in the presence and absence of Ca^2+^. The binding affinity (dissociation constant; *K*_d_) was determined to be 1.5 μM with Ca^2+^ and 0.88 μM without Ca^2+^. Notably, the *K*_d_ value without Ca^2+^ was 2-fold greater than that with Ca^2+^, indicating weaker binding of ERp57 to CNX in the presence of Ca^2+^ (Table 1). Thus, we concluded that the affinity of ERp57 for CNX is regulated by Ca^2+^.

### 2.3. Complex Formation between ERp57 and CNX Facilitates Oxidative Folding of HLA-Cw4 Heavy Chain

**Figure 3:**
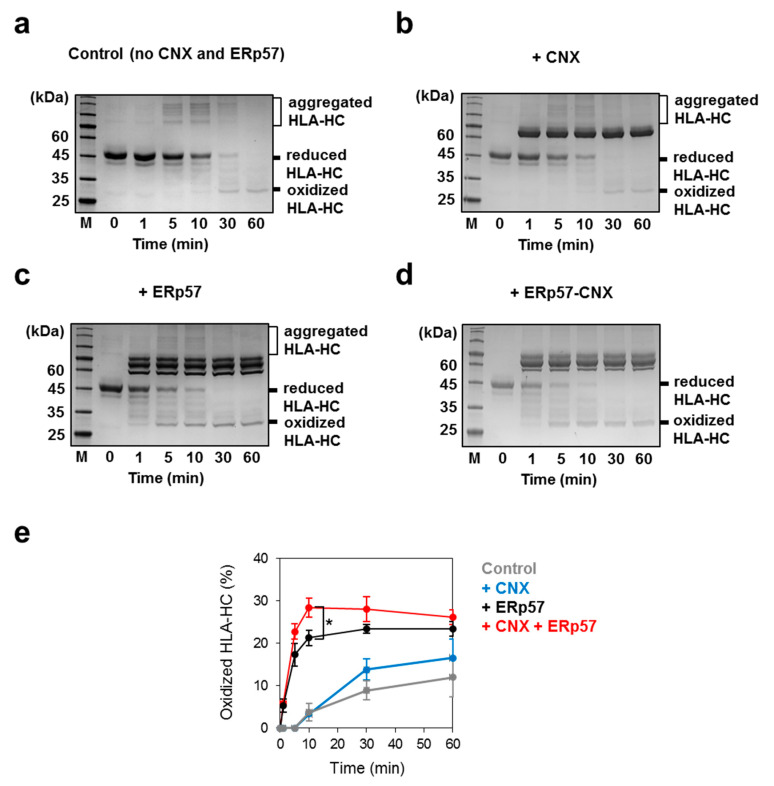
A mixture of ERp57 and CNX rapidly introduces disulfide bonds into HLA-Cw4. These panels show a time course of HLA-HC oxidation catalyzed by CNX and/or ERp57. Reduced and denatured HLA-Cw4 (5 µM) was incubated without CNX/ERp57 as a control (**a**), and with CNX (**b**), ERp57 (**c**), or CNX/ERp57 (**d**) under redox conditions (GSH:GSSG = 2 mM:1 mM). Reactions were quenched with 10 mM maleimide-PEG-2k at the selected time points and proteins were separated by non-reducing SDS-PAGE (10% polyacrylamide gels). Experiments were independently repeated three times with reproducible results. (**e**) Quantification and statistical analysis of the fraction of oxidized HLA-HC shown in Figure 3a–d. The relative band intensities of oxidized HLA-HC were quantified compared to the band intensity of reduced HLA-HC (*n* = 3, mean ± SD). * *p* < 0.05.

To gain insight into the enzymatic role of the ERp57-CNX complex, oxidative folding assays were carried out using HLA-HC as a substrate in non-reducing SDS-PAGE. HLA-Cw4 was prepared as inclusion bodies using an *Escherichia coli* expression system, and purified in the reduced and denatured state by reversed-phase high-pressure liquid chromatography (HPLC) with a Cosmosil 5C18-AR-II column. Fully reduced and denatured HLA-Cw4 contains four free cysteines (molecular weight 32 kDa), the alkylation (maleimide-PEG-2k) of which dramatically decreases the electrophoretic mobility (Figure 3a). Even when incubating under redox conditions with a reduced and oxidized glutathione (GSH:GSSG) ratio of 2 mM:1 mM at pH 8.0, very little reduced and denatured HLA-Cw4 (5 μM) formed disulfide bonded HC species, and instead formed higher molecular weight aggregates (Figure 3a). A mixture of ERp57 and CNX promptly introduced disulfide bonds into HLA-Cw4, although ERp57 or CNX alone converted reduced HLA-Cw4 into a fully oxidized form more slowly (Figure 3b–e). Notably, as was the case without chaperones, higher molecular weight HLA-Cw4 aggregates were observed at 5 and 10 min when CNX or ERp57 was added alone (Figure 3a–c). However, a mixture of ERp57 and CNX inhibited protein aggregation more effectively than CNX or ERp57 alone (Figure 3d), suggesting that complex formation regulates chaperone functions.

### 2.4. The ERp57-CNX Complex Inhibits Client Aggregation

**Figure 4:**
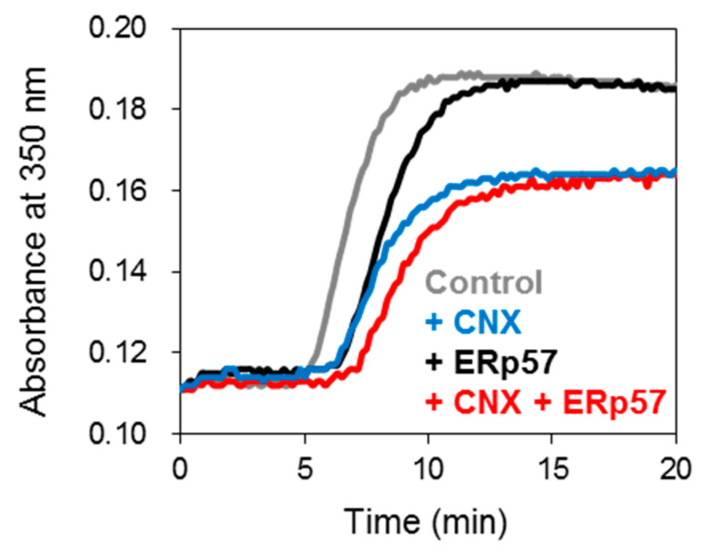
The ERp57-CNX complex inhibits client aggregation. Chaperone activity was assessed by monitoring the absorbance at 350 nm using 0.5 μM luciferase with or without ERp57 and/or CNX. The aggregation kinetics of luciferase with no chaperone, CNX, ERp57 and CNX-ERp57 are shown in grey, blue, black and red lines, respectively. Experiments were independently repeated three times with reproducible results.

To explore whether complex formation regulates chaperone functions, we probed protein aggregation using absorption spectroscopy. Luciferase was employed as a model substrate without disulfide bonds [29]. Thermal-induced aggregation of luciferase was observed by monitoring the increase in absorbance at 350 nm (Figure 4). Addition of ERp57 (Figure 4, red) yielded the same rate as spontaneous aggregation (black), suggesting that ERp57 has lower chaperone activity against amorphous aggregation than PDI and P5 [10,29]. Notably, only CNX or ERp57/CNX complex inhibited aggregation even more effectively than ERp57. This suggests that the ERp57 chaperone function is imparted by forming a complex with CNX. In other words, CNX likely promotes the anti-aggregation activity of ERp57 via Ca^2+^-regulated complex formation.

## 3. Discussion

**Figure 5:**
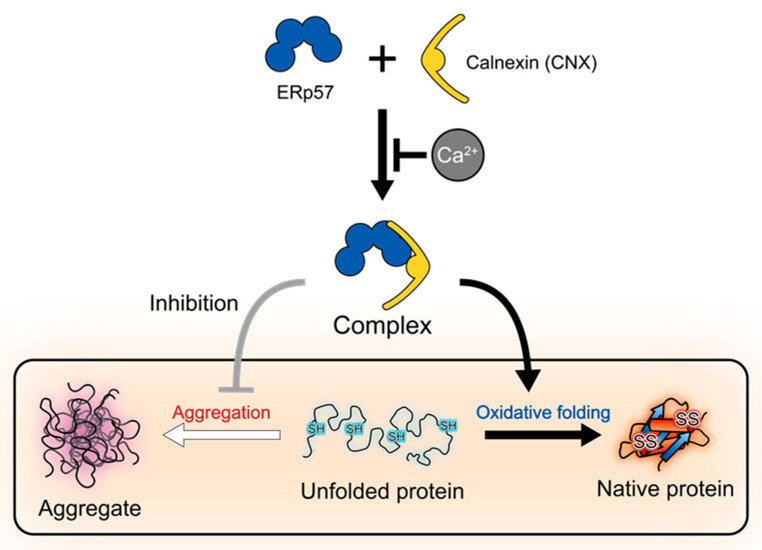
Proposed model of Ca^2+^-regulated ERp57-CNX complex formation promoting productive client folding.

In the present study, we demonstrated that the interaction between CNX and ERp57 is regulated by Ca^2+^ (Figure 1 and Figure 2). The complex tends to partially dissociate at 1 mM Ca^2+^, the concentration at which Ca^2+^ is stored in the ER. To maintain the Ca^2+^ concentration in the ER, sarco/endoplasmic reticulum Ca^2+^-ATPase (SERCA) enhances Ca^2+^ entry into the ER [30,31], and the inositol 1,4,5-triphosphate receptor (IP3R) releases Ca^2+^ from the ER [32]. Moreover, Ca^2+^ depletion is known to cause severe defects such as ER stress [33]. In this situation, aggregates of misfolded proteins accumulate in the ER lumen [34] and ER chaperone genes are transcriptionally induced to increase folding capacity via the unfolded protein response [35]. Therefore, much research has focused on ER-resident chaperones in response to Ca^2+^ concentration, and the present work demonstrates gain-of-function of enzymes/chaperones through depletion of Ca^2+^. Notably, the ERp57-CNX complex strongly promoted oxidative folding (Figure 3) and inhibited client aggregation (Figure 4), suggesting that this complex performs both enzymatic and chaperoning functions under abnormal physiological conditions such as Ca^2+^ depletion (Figure 5). Therefore, we believe that Ca^2+^-dependent ERp57-CNX complex formation is physiologically relevant and capable of responding to fluctuations in Ca^2+^ concentration. In line with this, previous studies demonstrated that the chaperoning functions of PDI and P5 are negatively suppressed by Ca^2+^ [10,23], implicating Ca^2+^ as a functional regulator of ER-resident chaperones.

ERp57 is known to bind not only to CNX but also CRT and ERp27 [36]. Regarding functional switching, nascent polypeptides co-translationally interact with the CNX-ERp57 complex, and partially folded clients post-translationally interact with the CRT-ERp57 complex [21]. Thus, a combination of ERp57 and partner chaperones appears to play a significant physiological role in the quality control of proteins inserted into the ER. A previous study demonstrated that ERp57 interacts with CRT under low Ca^2+^ conditions [27]. Consistent with this, our current results indicate that ERp57 also forms a complex with CNX in the absence of Ca^2+^ to control both enzymatic and chaperoning functions. Further studies should seek to clarify how ERp57 selects these partner proteins, which could help us to understand the detailed mechanism by which the functional relationship between ER-resident chaperone networks and Ca^2+^ ensures protein quality control in the ER.

## 4. Materials and Methods

### 4.1. Plasmid Construction

cDNA encoding human ERp57 (residues 25–505) and the luminal domain of human calnexin (CNX; residues 21–481) were subcloned into the *Nde*I and *Bam*HI sites of the pET15b vector (Novagen, Darmstadt, Germany). Plasmids encoded a 6-histidine tag at the N-terminus of the proteins. The 6-histidine tag in the CNX plasmid was removed and added at the C-terminus using a PrimeSTAR Mutagenesis Basal Kit (Takara Bio, Shiga, Japan) as described previously [37]. The plasmid for overexpression of HLA-Cw4 heavy chain in *E. coli* was prepared as previously described [38] with slight modification of removal of the biotinylation tag.

### 4.2. Recombinant Protein Expression and Purification

Recombinant human wild-type ERp57 and the luminal domain of CNX were overexpressed in *E. coli* strain BL21 (DE3) and purified as described previously [37,39]. Recombinant HLA-Cw4 heavy chain was overexpressed as inclusion bodies in the same cell type. Inclusion bodies were treated with 100 mM Tris/HCl (pH 8.0) buffer containing 8 M urea and 10 mM dithiothreitol. The proteins were purified by RP-HPLC (Hitachi High-Tech Corporation, Tokyo, Japan) using a Cosmosil 5C18-AR-II column (4.6 mm I.D. × 250 mm; Nacalai Tesque, Shiga, Japan) with monitoring at 220 nm and confirmation by mass spectrometry. The HLA-Cw4 heavy chain purified in the reduced/denatured state was lyophilized for storage.

### 4.3. Detection of ERp57-CNX Complex Formation by PAGE

CN-PAGE was performed using a modified version of the method of Pandhare et al. [28]. Purified wild-type ERp57 (2 μM) was incubated with CNX (2 μM) in buffer containing 50 mM Tris/HCl (pH 7.5), 0.05% (*w*/*v*) Coomassie Brilliant Blue (CBB) G-250, and 10% (*v*/*v*) glycerol in the absence or presence of 1 mM CaCl_2_ for 1 h at 30 °C to equilibrate intermolecular interactions. Samples were then incubated at 4 °C for 15 min, and separated by CN-PAGE or SDS-PAGE on 8% polyacrylamide gels prepared with WIDE RANGE Gel Preparation Buffer (Nacalai Tesque, Shiga, Japan) and stained with CBB G-250. For 2D-PAGE, parts of unstained gels from CN-PAGE were separated by second dimensional electrophoresis. Specifically, each lane was split into a gel strip, and the gel strip was incubated in SDS-sample buffer containing 5% 2-mercaptoethanol for 20 min at 60 °C. The gel strips were then subjected to reducing SDS-PAGE followed by staining with CBB G-250 [40]. The band intensities were analyzed by ImageJ (http://rsb.info.nih.gov/ij/index.html; accessed on 1 April 2021). Statistical analyses were performed using Student’s *t*-test.

### 4.4. Analysis of Binding by ITC

Wild-type ERp57 (370 μM) and CNX (25 μM) in 20 mM HEPES buffer (pH 7.2) containing 100 mM NaCl were prepared for ITC measurements in the absence or presence of 1 mM CaCl_2_. All samples were degassed for 3 min at 15 °C using a ThermoVac unit (Malvern Panalytical, Malvern, UK) before ITC measurements. ITC was carried out using a MicroCal VP-ITC instrument (Malvern Panalytical) at 15 °C. A solution of ERp57 in the syringe was titrated into a solution containing CNX in the cell via 28 injections at a constant interval of 600 s. The injection volume was 2 μL for the first injection and 10 μL for the remaining injections. The stirring speed of the syringe and the initial delay were set to 307 rpm and 600 s, respectively. Changes in the heat flow (i.e., ITC thermogram) were traced in real time with a 10 μcal s^−1^ reference power. After baseline correction and subtraction of the heat of dilution, the binding isotherms were fitted to the one-set-of-sites binding model incorporated in the MicroCal Origin software.

### 4.5. Oxidative Folding Assay of HLA-Cw4 Heavy Chain

Reduced/denatured HLA-Cw4 heavy chain (5 μM) was incubated with ERp57 (4 μM) and/or CNX (12 μM) in a redox buffer containing 100 mM Tris/HCl (pH 8.0), 2 mM GSH, 1 mM GSSG, 400 mM L-Arg, 1 mM EDTA, and 10% glycerol at 30 °C. At selected time points, the reaction was quenched with 2× SDS-sample buffer containing 10 mM maleimide-PEG-2k. All samples were separated by non-reducing SDS-PAGE followed by staining with CBB [39]. The band intensities were analyzed by ImageJ. The percentages of oxidized HLA-HC were calculated by dividing the band intensity of oxidized HLA-HC at each reaction time by that of reduced HLA-HC at 0 min. Statistical analyses were performed using Student’s *t*-test.

### 4.6. Chaperone Activity Assay of ERp57 and/or CNX

Luciferase (0.5 μM) as a model substrate was incubated with ERp57 (0.1 μM) and/or CNX (0.05 μM) in 50 mM HEPES buffer (pH 7.5). Luciferase aggregation was induced under agitation at 45 °C and monitored as turbidity at 350 nm using a SH-9000 microplate reader (Corona Electric Co., Ibaraki, Japan).

## Figures and Tables

**Table 1:** Thermodynamic parameters for ERp57 binding to CNX with/without Ca^2+^. *K*_d_, *H*, *T*, and *S* mean binding dissociation constant, enthalpy, temperature and entropy.

Sample	*K*_d_/μM	Δ*H*/kcal mol^−1^	*T*Δ*S*/kcal mol^−1^
ERp57 binding to CNX with Ca^2+^	1.52 ± 0.06	−3.81 ± 0.03	3.86 ± 0.53
ERp57 binding to CNX without Ca^2+^	0.88 ± 0.09	−3.31 ± 0.06	4.67 ± 0.12

## Data Availability

The authors declare that the data that support the findings of this study are available within the paper. All other information and samples are available from the corresponding author upon reasonable request.

## References

[1] Bulleid N.J., Ellgaard L. (2011). Multiple ways to make disulfides. Trends Biochem. Sci..

[2] Okumura M., Kadokura H., Inaba K. (2015). Structures and functions of protein disulfide isomerase family members involved in proteostasis in the endoplasmic reticulum. Free Radic. Biol. Med..

[3] Fass D., Thorpe C. (2018). Chemistry and Enzymology of Disulfide Cross-Linking in Proteins. Chem. Rev..

[4] Matsusaki M., Kanemura S., Kinoshita M., Lee Y.-H., Inaba K., Okumura M. (2020). The Protein Disulfide Isomerase Family: From proteostasis to pathogenesis. Biochim. Biophys. Acta Gen. Subj..

[5] Hatahet F., Ruddock L.W. (2009). Protein Disulfide Isomerase: A Critical Evaluation of Its Function in Disulfide Bond Formation. Antioxid. Redox Signal..

[6] Wang C., Li W., Ren J., Fang J., Ke H., Gong W., Feng W., Wang C.-C. (2013). Structural Insights into the Redox-Regulated Dynamic Conformations of Human Protein Disulfide Isomerase. Antioxid. Redox Signal..

[7] Okumura M., Noi K., Kanemura S., Kinoshita M., Saio T., Inoue Y., Hikima T., Akiyama S., Ogura T., Inaba K. (2019). Dynamic assembly of protein disulfide isomerase in catalysis of oxidative folding. Nat. Chem. Biol..

[8] Sato Y., Kojima R., Okumura M., Hagiwara M., Masui S., Maegawa K.-I., Saiki M., Horibe T., Suzuki M., Inaba K. (2013). Synergistic cooperation of PDI family members in peroxiredoxin 4-driven oxidative protein folding. Sci. Rep..

[9] Kojima R., Okumura M., Masui S., Kanemura S., Inoue M., Saiki M., Yamaguchi H., Hikima T., Suzuki M., Akiyama S. (2014). Radically Different Thioredoxin Domain Arrangement of ERp46, an Efficient Disulfide Bond Introducer of the Mammalian PDI Family. Structure.

[10] Okumura M., Kanemura S., Matsusaki M., Kinoshita M., Saio T., Ito D., Hirayama C., Kumeta H., Watabe M., Amagai Y. (2021). A unique adhesive motif of protein disulfide isomerase P5 supports its function via dimerization. Structure.

[11] Okumura M., Noi K., Inaba K. (2021). Visualization of structural dynamics of protein disulfide isomerase enzymes in catalysis of oxidative folding and reductive unfolding. Curr. Opin. Struct. Biol..

[12] Dong G., Wearsch P.A., Peaper D.R., Cresswell P., Reinisch K.M. (2009). Insights into MHC Class I Peptide Loading from the Structure of the Tapasin-ERp57 Thiol Oxidoreductase Heterodimer. Immunity.

[13] Oliver J.D., Van Der Wal F.J., Bulleid N.J., High S. (1997). Interaction of the Thiol-Dependent Reductase ERp57 with Nascent Glycoproteins. Science.

[14] Oliver J.D., Roderick H.L., Llewellyn D.H., High S. (1999). ERp57 Functions as a Subunit of Specific Complexes Formed with the ER Lectins Calreticulin and Calnexin. Mol. Biol. Cell.

[15] Molinari M., Eriksson K.K., Calanca V., Galli C., Cresswell P., Michalak M., Helenius A. (2004). Contrasting Functions of Calreticulin and Calnexin in Glycoprotein Folding and ER Quality Control. Mol. Cell.

[16] Ruddock L.W., Molinari M. (2006). N-glycan processing in ER quality control. J. Cell Sci..

[17] Rutkevich L.A., Williams D.B. (2011). Participation of lectin chaperones and thiol oxidoreductases in protein folding within the endoplasmic reticulum. Curr. Opin. Cell Biol..

[18] Pollock S., Kozlov G., Pelletier M.-F., Trempe J.-F., Jansen G., Sitnikov D., Bergeron J.J.M., Gehring K., Ekiel I., Thomas D.Y. (2004). Specific interaction of ERp57 and calnexin determined by NMR spectroscopy and an ER two-hybrid system. EMBO J..

[19] Kozlov G., Maattanen P., Schrag J.D., Pollock S., Cygler M., Nagar B., Thomas D.Y., Gehring K. (2006). Crystal Structure of the bb′ Domains of the Protein Disulfide Isomerase ERp57. Structure.

[20] Maenaka K., Jones E.Y. (1999). MHC superfamily structure and the immune system. Curr. Opin. Struct. Biol..

[21] Farmery M.R., Allen S., Allen A.J., Bulleid N.J. (2000). The Role of ERp57 in Disulfide Bond Formation during the Assembly of Major Histocompatibility Complex Class I in a Synchronized Semipermeabilized Cell Translation System. J. Biol. Chem..

[22] Blees A., Januliene D., Hofmann T., Koller N., Schmidt C., Trowitzsch S., Moeller A., Tampé R. (2017). Structure of the human MHC-I peptide-loading complex. Nat. Cell Biol..

[23] Primm T.P., Walker K.W., Gilbert H.F. (1996). Facilitated Protein Aggregation. J. Biol. Chem..

[24] Wada I., Rindress D., Cameron P.H., Ou W.J., Doherty J.J., Louvard D., Bell A.W., Dignard D., Thomas D.Y., Bergeron J.J. (1991). SSR alpha and associated calnexin are major calcium binding proteins of the endoplasmic reticulum membrane. J. Biol. Chem..

[25] Meldolesi J., Pozzan T. (1998). The endoplasmic reticulum Ca^2+^ store: A view from the lumen. Trends Biochem. Sci..

[26] Thammavongsa V., Mancino L., Raghavan M. (2005). Polypeptide Substrate Recognition by Calnexin Requires Specific Conformations of the Calnexin Protein. J. Biol. Chem..

[27] Corbett E.F., Oikawa K., Francois P., Tessier D.C., Kay C., Bergeron J.J.M., Thomas D.Y., Krause K.-H., Michalak M. (1999). Ca^2+^ Regulation of Interactions between Endoplasmic Reticulum Chaperones. J. Biol. Chem..

[28] Pandhare A., Stuebler A.G., Pirayesh E., Jansen M. (2019). A modified clear-native polyacrylamide gel electrophoresis technique to investigate the oligomeric state of MBP-5-HT3A-intracellular domain chimeras. Protein Expr. Purif..

[29] Okumura M., Kadokura H., Hashimoto S., Yutani K., Kanemura S., Hikima T., Hidaka Y., Ito L., Shiba K., Masui S. (2014). Inhibition of the Functional Interplay between Endoplasmic Reticulum (ER) Oxidoreduclin-1α (Ero1α) and Protein-disulfide Isomerase (PDI) by the Endocrine Disruptor Bisphenol A. J. Biol. Chem..

[30] Inoue M., Sakuta N., Watanabe S., Zhang Y., Yoshikaie K., Tanaka Y., Ushioda R., Kato Y., Takagi J., Tsukazaki T. (2019). Structural Basis of Sarco/Endoplasmic Reticulum Ca^2+^-ATPase 2b Regulation via Transmembrane Helix Interplay. Cell Rep..

[31] Zhang Y., Inoue M., Tsutsumi A., Watanabe S., Nishizawa T., Nagata K., Kikkawa M., Inaba K. (2020). Cryo-EM structures of SERCA2b reveal the mechanism of regulation by the luminal extension tail. Sci. Adv..

[32] Bosanac I., Alattia J.-R., Mal T.K., Chan J., Talarico S., Tong F.K., Tong K.I., Yoshikawa F., Furuichi T., Iwai M. (2002). Structure of the inositol 1,4,5-trisphosphate receptor binding core in complex with its ligand. Nat. Cell Biol..

[33] Lytton J., Westlin M., Hanley M. (1991). Thapsigargin inhibits the sarcoplasmic or endoplasmic reticulum Ca-ATPase family of calcium pumps. J. Biol. Chem..

[34] Rao R.V., Bredesen D.E. (2004). Misfolded proteins, endoplasmic reticulum stress and neurodegeneration. Curr. Opin. Cell Biol..

[35] Lee A.-H., Iwakoshi N.N., Glimcher L.H. (2003). XBP-1 Regulates a Subset of Endoplasmic Reticulum Resident Chaperone Genes in the Unfolded Protein Response. Mol. Cell. Biol..

[36] Alanen H.I., Williamson R.A., Howard M.J., Hatahet F.S., Salo K.E.H., Kauppila A., Kellokumpu S., Ruddock L.W. (2006). ERp27, a New Non-catalytic Endoplasmic Reticulum-located Human Protein Disulfide Isomerase Family Member, Interacts with ERp57. J. Biol. Chem..

[37] Urade R., Okudo H., Kato H., Moriyama T., Arakaki Y. (2004). ER-60 Domains Responsible for Interaction with Calnexin and Calreticulin. Biochemistry.

[38] Shiroishi M., Tsumoto K., Amano K., Shirakihara Y., Colonna M., Braud V.M., Allan D.S.J., Makadzange A., Rowland-Jones S., Willcox B. (2003). Human inhibitory receptors Ig-like transcript 2 (ILT2) and ILT4 compete with CD8 for MHC class I binding and bind preferentially to HLA-G. Proc. Natl. Acad. Sci. USA.

[39] Kanemura S., Sofia E.F., Hirai N., Okumura M., Kadokura H., Inaba K. (2020). Characterization of the endoplasmic reticulum–resident peroxidases GPx7 and GPx8 shows the higher oxidative activity of GPx7 and its linkage to oxidative protein folding. J. Biol. Chem..

[40] Matsusaki M., Okuda A., Masuda T., Koishihara K., Mita R., Iwasaki K., Hara K., Naruo Y., Hirose A., Tsuchi Y. (2016). Cooperative Protein Folding by Two Protein Thiol Disulfide Oxidoreductases and ERO1 in Soybean. Plant Physiol..

